# Implications of purinergic receptor-mediated intracellular calcium transients in neural differentiation

**DOI:** 10.1186/1478-811X-11-12

**Published:** 2013-02-17

**Authors:** Talita Glaser, Rodrigo R Resende, Henning Ulrich

**Affiliations:** 1 Departamento de Bioquímica, Instituto de Química, Universidade de São Paulo, Avenida Professor Lineu Prestes, 748, São Paulo, CEP 05508-900, Brazil; 2 Departamento de Bioquímica e Imunologia, Instituto de Ciências Biológicas, Universidade Federal de Minas Gerais, Belo Horizonte, Brazil

**Keywords:** Purinergic receptors, Intracellular calcium transients, Neural differentiation, Neural fate specification

## Abstract

Purinergic receptors participate, in almost every cell type, in controlling metabolic activities and many physiological functions including signal transmission, proliferation and differentiation. While most of P2Y receptors induce transient elevations of intracellular calcium concentration by activation of intracellular calcium pools and forward these signals as waves which can also be transmitted into neighboring cells, P2X receptors produce calcium spikes which also include activation of voltage-operating calcium channels. P2Y and P2X receptors induce calcium transients that activate transcription factors responsible for the progress of differentiation through mediators including calmodulin and calcineurin. Expression of P2X2 as well as of P2X7 receptors increases in differentiating neurons and glial cells, respectively. Gene expression silencing assays indicate that these receptors are important for the progress of differentiation and neuronal or glial fate determination. Metabotropic receptors, mostly P2Y1 and P2Y2 subtypes, act on embryonic cells or cells at the neural progenitor stage by inducing proliferation as well as by regulation of neural differentiation through NFAT translocation. The scope of this review is to discuss the roles of purinergic receptor-induced calcium spike and wave activity and its codification in neurodevelopmental and neurodifferentiation processes.

## 

The importance of establishing cellular replacement therapies is increasing nowadays. These therapies are based on stem cells due to their capability of originating other cell types by differentiation, or even releasing factors that can reduce inflammatory responses and promote homing of adult stem cells of the proper patient to the site of injury. However, the molecular mechanisms of development, cell differentiation, successful cell engraftment and recruitment of endogenous stem cells in case of injury need yet to be resolved. Thus, major efforts are being undertaken in order to understand the process of neural differentiation, mediated by intracellular signaling triggered by external and internal stimuli, resulting in differential gene transcription pattern and neural phenotype determination.

## Intracellular calcium signaling and purinergic receptors

Among different pathways coordinating intracellular signaling, the most prominent is intracellular calcium signaling (ICS), controlling various cellular processes including proliferation, motility, apoptosis and differentiation [1]. ICS is impressively diverse and consists of mechanisms that differ in frequency, amplitude and spatio-temporal patterning depending on an extensive molecular repertoire of signaling components. The free intracellular calcium concentration ([Ca^2+^_i_) of a resting cell is in the range of 10–100 nM. Following physiological stimulation, [Ca^2+^_i_ levels can rise up to 1-2 μM concentrations. ICS is codified by the peak amplitude and frequency of [Ca^2+^_i_ transients, promoted by the entry of external Ca^2+^ through Ca^2+^ channels or the release of Ca^2+^ from internal stores. These internal stores are deposited within internal membrane structures such as the endoplasmic reticulum (ER). Following activation of G-protein-coupled receptors, phospholipase C-β (PLC-β) cleaves phosphatidylinositol 4,5-bisphosphate, releasing diacylglycerol and inositol-1,4,5-trisphosphate (IP3) which diffuses into the cell for activation of IP3 receptors (IP3R) and releasing Ca^2+^ from the ER. Moreover, Ca^2+^ enters the cytosol and activates ryanodine receptor (RYR) channels following activation of voltage-operated channels (VOCs), or receptor-operated channels (ROCs); this process is called Ca^2+^-induced Ca^2+^ release [2-4].

There are mainly two types of spontaneous intracellular Ca^2+^ transients: waves and spikes. The first one is mediated by IP3R and/or RYR activation and involves sensitivity to [Ca^2+^_i_ levels. This Ca^2+^ entry pathway is active at resting potential and is amplified by Ca^2+^ release from intracellular stores, and increases [Ca^2+^_i_ to a lower level than that attained by spikes. In the presence of gap junctions connecting cells, these intracellular waves can spread to neighboring cells, thereby coordinating neural activity and physiological processes of many cells [5,6]. Compared to Ca^2+^ spikes, waves reveal a lower frequency with a mean duration of more than 30s, as observed in growth cones; their generation does not depend on action potentials. Waves occur locally and decay with distance from the site of initiation.

Calcium spikes depend on Ca^2+^ influx through VOCs or ROCs and Ca^2+^ release from intracellular stores (Ca^2+^ induced Ca^2+^ release via RYR), and achieve mean [Ca^2+^]_i_ levels of 500 nM. They are characterized by their frequency (mean duration approximately 10s), and occur throughout an excitable cell, since they involve Ca^2+^-dependent action potentials.

Spontaneous Ca^2+^ spike frequency in cultured neurons initially varies from 1-10/h and then declines. Similar patterns of spike activity were observed in neural tube stages *in vivo*[2,7] and during neuronal differentiation of embryonal carcinoma (CSC, a model for pluripotent embryonic stem cells) and adult bone marrow mesenchymal (hMSC) stem cells [8,9]. Notably, these low frequencies of calcium transients regulate gene transcription and are suggested to be essential for the progress of neural differentiation and phenotype specification [2,10-12].

Chemical and electrical signals, mediated by metabotropic and ionotropic receptors and VOCs promote intracellular calcium signaling and subsequent induction of differentiation. Adenosine 5’-triphosphate (ATP)-activated metabotropic and ionotropic receptors, also denominated as purinergic receptors, have drawn a lot of attention, due to their wide expression in almost every cell including stem cells. These receptors belong to the first neurotransmitter receptors expressed during development [13,14]. ATP is the mainly purinergic messenger molecule and is released from cells in physiological conditions by exocytosis, transporters or even lysosomes. When the release occurs by damaged cells in an uncontrolled manner, ATP contributes to cell death and disease states. Once released into the extracellular space, ATP is degraded by ectonucleotidases producing the signaling molecules adenosine diphosphate (ADP), adenosine monophosphate [15] and adenosine [14]. Based on pharmacological and structural properties, purinergic receptors are divided into metabotropic P1 and P2Y receptors as well as P2X ionotropic receptors.

P1 receptors subtypes are selective for adenosine and are classical seven-transmembrane metabotropic receptors coupled to several families of Gi, Go and Gs proteins. There are four types of adenosine receptors (A1, A2A, A2B and A3) differing by pharmacological and functional properties [15]. A1 and A3 receptors exert inhibitory effects on adenylyl cyclase activity (mediated through Gi/o proteins) and also regulate PLC-β activity and thus IP3 synthesis [14]. P2 receptor subtypes are activated by ATP, ADP, uridine-5'-triphosphate (UTP), uridine-5'-diphosphate (UDP) or UDP-glucose. P2 receptors are further divided into P2X and P2Y subtypes based on their structural characteristics [13].

P2X receptors as ATP-gated cationic (Na^+^/K^+^/Ca^2+^) channels [13,16], are assembled in trimeric form as homomeric or heteromeric receptors from seven subunits (P2X1-P2X7). Recombinant P2X1, P2X2, P2X3, P2X4, P2X2/3, P2X2/6, P2X4/6, P2X1/5 as well as P2X7 receptors, when activated in a silent environment of cells not expressing any endogenous purinergic receptor, were all shown to be permeable for Ca^2+^[17]. P2X receptors are mostly expressed by excitable cells, and Ca^2+^ entry through P2X receptor channels provides an important regulation mechanism of physiological responses *in vivo*, while aberrant Ca^2+^ entry, mostly mediated by P2X7 receptors, is suggested to be involved in pathophysiological conditions such as cell death, neuroinflammation and excitotoxic brain damage during epilepsy [18-22].

Metabotropic P2Y purinergic receptors activated by ATP, ADP, UTP, UDP or UDP glucose are composed by P2Y1,2,4,6,11,12,13,14 subtypes based on phylogenetic similarity [14]. P2Y1,2,4,6,11 subtypes are coupled to Gq/G11 proteins activating PLC-β, thus inducing IP3-mediated Ca^2+^ release from the ER [13,14,23]. P2Y12,13,14 receptors inhibit adenylyl cyclase activity via Gi/o proteins. These latter-mentioned receptors also participate in regulating [Ca^2+^_i_ levels. For instance, P2Y receptor subtypes acting via Gi/o proteins can regulate N-type Ca^2+^ channel activity [24-26]. P2Y receptors are expressed in the central and autonomic nervous systems as well as by most non-excitatory cells where they exert long-term effects by regulating crucial cellular functions including proliferation and differentiation [14].

Recent studies have focused on changes of expression patterns and functions of this receptor family during differentiation from embryonic into neuronal cells [8,27]. The developmental fate of differentiating stem cells depends on the ‘niche’, in which the cells exist, and several associated signaling systems have been pinpointed [28,29]. In this review, we discuss the importance of ICS for the differentiation process of stem cells into neural cells with special emphasis on purinergic receptor function during Ca^2+^ signaling.

## Purinergic receptors triggering ICS in the nervous system and neural differentiation

**Figure 1:**
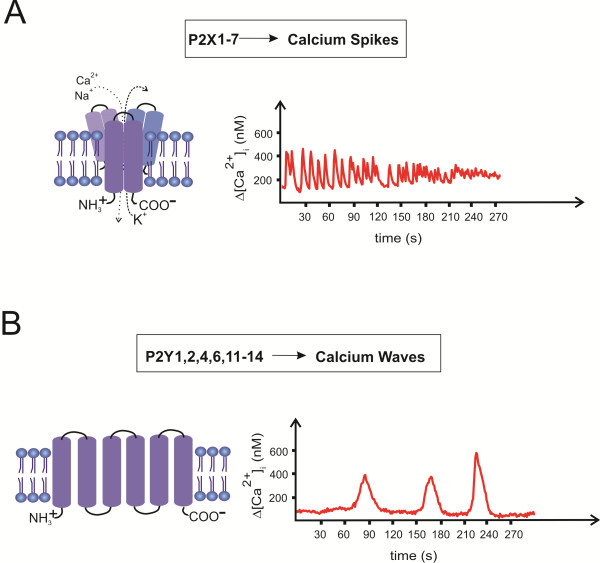
**Oscillations of [Ca**^**2+**^**]**_**i **_**levels and purinergic receptors.** Ionotropic P2X receptors trigger calcium spikes characterized by amplitudes and frequency (**A**), while metabotropic P2Y receptors induce calcium waves with lower amplitudes and frequencies (**B**). P2X receptors are composed of three subunits, with two transmembrane loops each. G protein-coupled metabotropic P2Y receptors are composed of 7 transmembrane loops.

Many of the signal transduction pathways that control cell metabolism, survival and differentiation are activated by elevation of [Ca^2+^_i_ levels following activation of purinergic receptors. Moreover, numerous experimental data point at essential functions of purinergic signaling in neural differentiation and brain development. It has been well established that purinergic receptor activation triggers [Ca^2+^_i_ transients that are involved in developmental processes of the embryo [30,31]. Pioneering studies of Nicholas Spitzer that the progress of neurogenesis and phenotype determination, such as neurotransmitter specification, is encoded by naturally occurring patterns of [Ca^2+^_i_ transients, are in agreement with essential functions of purinergic receptor signaling for cortex development. As detailed above, P2X receptors by inducing Ca^2+^ influx generate repetitive [Ca^2+^_i_ transients in spike form with activation of RYR while P2Y receptors act through IP3-induced intracellular Ca^2+^ release which then is propagated in wave form (Figure 1).

Ionotropic purinergic receptors also participate in regulation of neural proliferation and neurogenesis by inducing calcium spikes. In agreement, P2X2 and P2X6 receptor subunit expression was enhanced together with the enrichment of neurons during differentiation of rat embryonic telencephalon [32]. For instance, P2X receptor activity is suggested to be involved in hippocampal neurogenesis by inducing proliferation of hippocampal progenitor cells [33]. The P2X7 receptor usually participates in pore formation, but its abundant presence in synaptic structure suggests a role in synaptic plasticity establishment [26]. Some experimental data indicate that P2X7 receptors, rather than connexin-hemichannels, mediate ATP release and amplification of astrocytic intercellular Ca^2+^ signaling [34]. In view of that, it will be worth to study the participation of P2X7 receptors in directing of migration and neurogenesis, such as shown for P2Y1 receptors in radial glial cells during cortex development [6].

**Figure 2:**
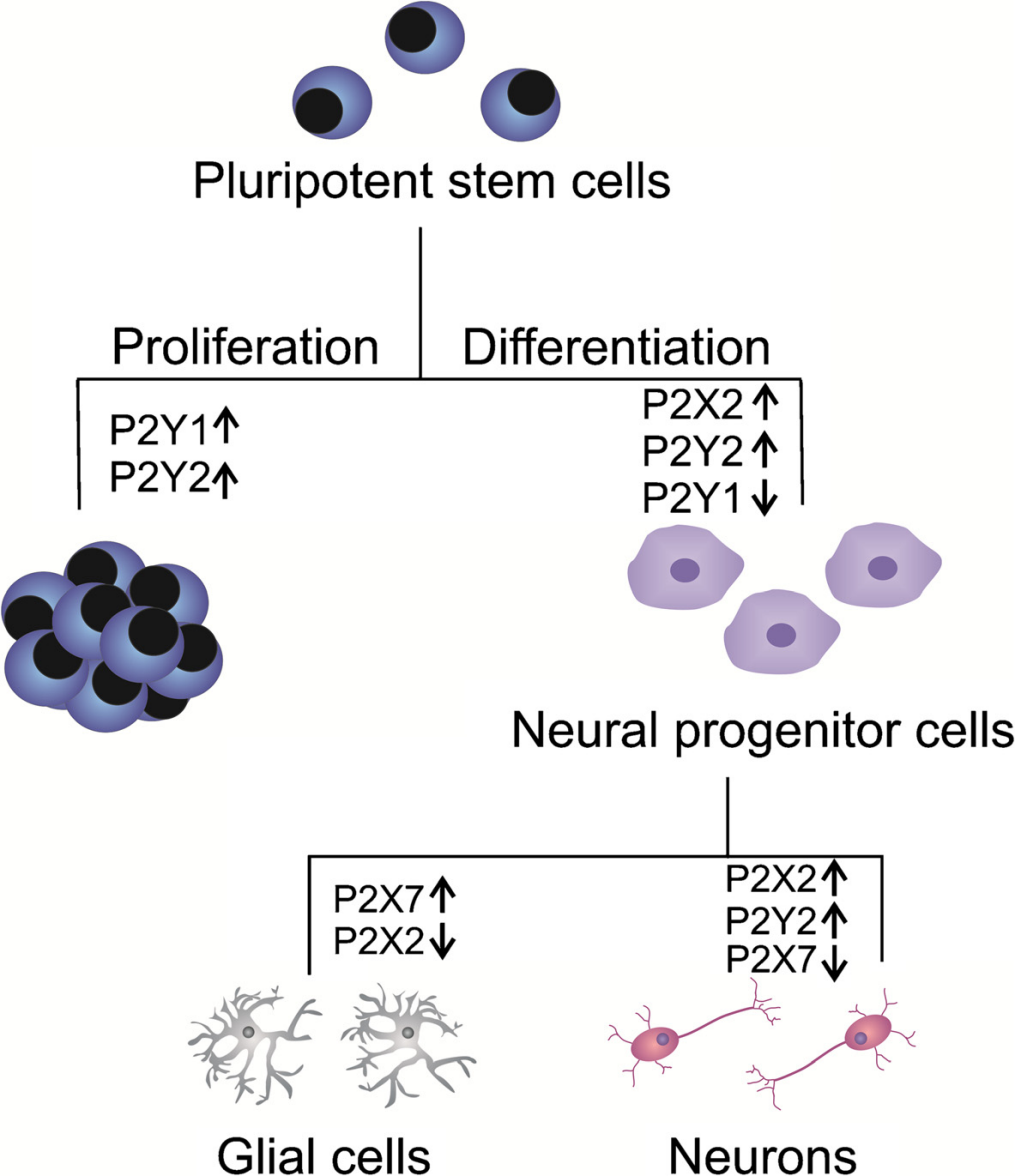
**Functions of P2X and P2Y receptor subtypes in stem cell biology.** P2Y1 and P2Y2 receptors regulate proliferation of pluripotent stem cells such as studied using P19 embryonal carcinoma cells as *in vitro* model. P2Y2 and P2Y1 receptors promote proliferation and neural differentiation of pluripotent and neural progenitor cells. P2Y2, P2X2 and P2X7 receptors participate in later differentiation and neural phenotype determination. P2X2 and P2X7 receptor expression/activity levels provide a further switch for neuronal or glial fate of P19 cell differentiation. Arrows indicate increases or decreases in receptor expression and activity levels in respective stages of differentiation [36].

Ionotropic purinergic receptors were also involved in the progress of pluripotent P19 CSC differentiation and neural phenotype determination. Functions of P2X2 and P2X7 receptor subtypes in conditions of down-regulation of receptor gene expression were studied by stable RNA interference. Knock-down of P2X2 receptor expression along neural differentiation resulted in diminished expression of β-3-tubulin expression indicating interference with the progress of neurogenesis. On the other hand, P2X7 receptor expression and activity was related to induction of proliferation and gliogenesis, since permanent P2X7 receptor RNA interference resulted in reduced 5’-bromo-2'-deoxyuridine (BrdU) incorporation and glial fibrillary acidic (GFAP) protein expression [35] (see Figure 2 for a comprehensive scheme of metabotropic and ionotropic purinergic receptor implication in proliferation and differentiation induction of P19 CSC).

Our laboratory has shown that a full-length and an alternatively spliced form of the mouse P2X6 receptor gene are expressed in mouse P19 CSC, an *in vitro* model for early neuroectodermal differentiation. The truncated alternatively spliced form was present at the undifferentiated stage of P19 CSC, and was predominant compared to the full-length form during the whole course of neuronal differentiation of these cells [37] suggesting that splicing could provide a mechanism for regulation of P2X6 subunit expression and formation of functional P2X receptors with P2X6 subunit contribution.

The involvement of these receptors in ATP-induced [Ca^2+^]_i_ transients was probed in pharmacological studies. Moreover, embryonic P19 CSC expressed various other functional subtypes including P2Y1, P2Y2 and P2X4 receptors or P2X-heteromultimeric receptors. In neuronal-differentiated cells, P2Y2, P2Y6, P2X2 and possibly P2X2/P2X6 heteromeric receptors were the major mediators of purinergic receptor-mediated [Ca^2+^]_i_ elevations.

P2Y1 receptor activation produces [Ca^2+^_i_ transients which are then propagated in wave form through neighbouring cells by gap junctions and connexin 43-hemichannels resulting in cell cycle synchronization of migrating neural progenitors and radial glia cells in the subventricular zone for cortex development [6]. ATP has also been shown to induce proliferation of human neural stem cells (NSC) cultured from telencephalon tissues from a 15-week gestational age embryo [38]. P2Y1 receptor-mediated [Ca^2+^_i_ transients resulted in Ca^2+^/calmodulin (CAM)-dependent protein kinase II (CaMKII) activation in cell soma and neurites of cerebellar granule neurons, followed by cAMP/Ca^2+^ response element binding protein (CREB) phosphorylation and modulation of gene transcription [39]. Neurotrophic effects, such as observed in Neuro2A cells, were induced by P2Y1 receptor signaling [40]. Here, low-frequency global and local Ca^2+^ transients induced by purinergic receptor activation during early stages of differentiation of neural progenitor cells promoted neurite outgrowth and the onset of GABAergic neurotransmitter phenotype specification. Surprisingly, spontaneous Ca^2+^ signals in individual precursors were not synchronized with Ca^2+^ transients in surrounding cells, indicating the existence of a different pathway, not depending on connexin 43-hemichannel-mediated intercellular Ca^2+^ signaling [41].

Calcium ions also plays an important role in proliferation and differentiation of hMSCs. Spontaneous [Ca^2+^_i_ oscillations occur without agonist stimulation in hMSCs. These [Ca^2+^_i_ transient are mediated by IP3-induced Ca^2+^ release and controlled by an autocrine/paracrine signaling pathway in which ATP is secreted via a hemi-gap junction channel and then stimulates the P2Y_1_ receptor, resulting in the activation of PLC-β for IP3 production. Furthermore, [Ca^2+^_i_ oscillations are associated with nuclear factor of activated T-cell (NFAT) translocation into the nucleus of undifferentiated hMSCs, providing a new role for [Ca^2+^_i_ oscillations in such stem cells [42].

The P2Y2 receptor subtype, another purinergic receptor involved in neural differentiation, which activates of PLC-β, intracellular Ca^2+^ release and intercellular Ca^2+^ waves, important for embryonic development [43]. However in neural stem cells, Lin and coworkers [44] described that neural progenitor proliferation is modulated by an autocrine loop. These cells release ATP and thus activate P2Y receptors for proliferation maintenance. Blockade of proliferation and induction to neural differentiation occurred only when purinergic receptor activity had been antagonized and [Ca^2+^_i_ transients had diminished.

In undifferentiated P19 CSC, ATP provoked acceleration of proliferation via P2Y1 and P2Y2 receptor activation. P19 CSC that progressed to the progenitor stage revealed down-regulated P2Y1 receptor expression, while activation of IP3-sensitive intracellular Ca^2+^ stores was mediated by P2Y2 receptors. The progress of neuronal differentiation and phenotype transition was determined by analysis of nestin and neuron-specific enolase gene and protein expression levels [27,45].

## Activation of transcription factors by ICS

It is important to highlight that during differentiation many immediate early genes are activated in order to regulate cell’s genomic responses to environmental stimuli. Underlying intracellular mechanisms are not clear yet; however, Sheng and coworkers showed that c-fos expression, an immediate early gene, depends on calcium influx. This increase activates the calcium response element (CaRE) and thus results in phosphorylation/activation of the CaRE binding protein and consequently c-fos transcription [46].

The transcription factor CREB is activated in neurons in response to trans-synaptic signaling and regulates the expression of genes important for adaptive neuronal responses, such as behavioral adaptation to changes in the environment [47], as well as for more complex neural functions, such as learning and memory formation [48]. Target genes include immediate early genes, such as c-fos [46], and molecules essential for synaptic function, including brain derived neurotrophic factor (BDNF) [49,50] and neuronal nitric oxide synthase (nNOS) [51]. In addition to its functions in mature neurons, CREB regulates cell proliferation, differentiation, and survival responses in a range of cell types in developing vertebrates [52-54]. CREB is inactive as a transcription factor until a cell is exposed to any one of a range of extracellular stimuli that trigger CREB phosphorylation at a specific site. Ser133 within its kinase-inducible domain promotes association of CREB with a co-adaptor protein, the CREB binding protein (CBP). The recruitment of CBP by CREB to the promoter of a CREB target gene then induces the assembly of an active polymerase II transcription complex, thus leading to target gene activation [55]. The kinetics of CREB Ser142 and Ser143 phosphorylation suggest that, when both of these phosphorylation events occur together with Ser133 phosphorylation, they promote CREB activation [56]. Moreover, experimental evidence indicates that Ca^2+^/CaM dependent kinase IV (CaMKIV) and CREB play a critical role in mediating calcium-induced dendritic growth in cortical neurons. A constitutively active form of CaMKIV induces dendritic growth in the absence of extracellular stimulation and activates the transcription factor CREB [57].

In hippocampal neurons, signaling to CREB activation can be triggered by elevations in nuclear calcium concentration alone and does not require import of cytoplasmic proteins into the nucleus. The nucleus is particularly suited to integrate neuronal firing patterns, and specifies the transcriptional outputs through a burst frequency and nuclear calcium amplitude conversion. Calcium release from intracellular stores promotes calcium wave propagation into the nucleus, which is critical for CREB-mediated transcription by synaptic receptors. Pharmacological modulation of nuclear calcium or modulation of gene expression levels of proteins involved in this process may directly affect stem cell differentiation during development [58].

**Figure 3:**
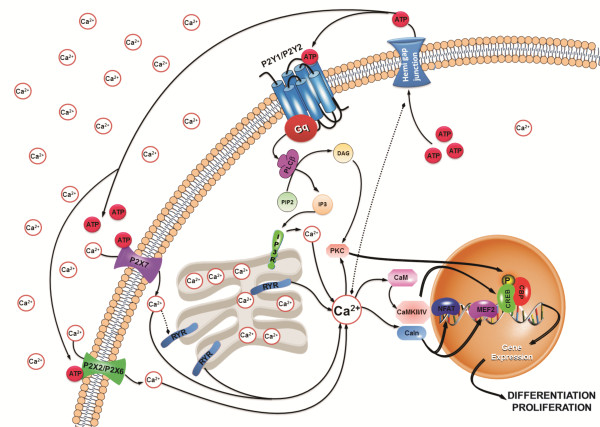
**P2X and P2Y receptor-mediated calcium signal transduction for neural differentiation and proliferation.** Activation of ionotropic P2X and metabotropic P2Y receptors trigger increases of [Ca^2+^]_i_ by promoting influx of extracellular calcium or by endoplasmatic reticulum calcium release, respectively. These [Ca^2+^]_i_ transients induce neural gene expression, by activation of some kinases or phosphatases which then stimulate transcription factors such as CREB, MEF2 and NFAT. Abbreviations: IP3, inositol 1,4,5-trisphosphate. RYR, ryanodine receptor. Gq, Gq protein. PLCβ, phospholipase C-β. PIP2, phosphatidylinositol 4,5-bisphosphate. DAG, diacylglycerol. IP3R, IP3 receptor. CaMK, Ca^2+^/calmodulin-dependent protein kinase. CREB, cAMP/Ca^2+^ response-binding element. CBP, CREB-binding protein. MEF2, myocyte enhancer factor-2. NFAT, nuclear factor of activated T-cells. PKC, protein kinase C. CaM, calmodulin. Caln, calcineurin.

Another important regulatory molecule that is sensible to changes in [Ca^2+^_i_ is the transcription factor myocyte enhancer factor-2 (MEF2), which is highly expressed in neurons and during embryogenesis. Experimental evidence indicates roles for MEF2 as a calcium-dependent regulator of neuronal differentiation and function. The calcium-binding protein CaM is activated by signals that trigger rises in [Ca^2+^_i_ resulting in Ca^2+^-bound CaM association with calcineurin (Cn), and thus releases Cn from its repressive effects. Cn dephosphorylates NFAT and MEF2, allowing them to translocate into the nucleus and consequently switch on gene transcription [59]. When Ca^2+^ entry is prevented or Cn activity is inhibited, NFAT is rephosphorylated by NFAT kinases and rapidly leaves the nucleus (t1/2 ∼ 15 min), and NFAT-dependent gene expression is terminated [60-63]. As a result of this absolute dependence on Ca^2+^/Cn signaling, NFAT has a remarkable ability to sense dynamic changes in intracellular Ca^2+^ levels and frequencies of Ca^2+^ oscillations in cells [64] (Figure 3). Furthermore, activation of CaMKII, through high-amplitude calcium spikes [65], induces neural gene expression through transcription factors of the MEF2 family [59].

Gene expression induced by [Ca^2+^_i_ transients, triggered by activation of VOCs and [66] and purinergic P2X or P2Y receptors, could comprise general regulation mechanisms of neural differentiation. Such hypothesis is in agreement with the observation that purinergic signaling is present and critical in switching on genes for development of the nervous system and the eye of the *Xenopus* around the time of gastrulation [67-69]. Ca^2+^ waves occur during early development, between stages 9 and 12 in the dorsal ectoderm of *Xenopus*[70]. From stage 10 onwards, [Ca^2+^_i_ transients are limited to regions of forebrain, midbrain and eyes overlapping with the approximate location and time of ATP release [69,71]. The relationship between P2 receptor and transcription factor activation by ICS is well illustrated in Figure 3.

## Conclusions

ICS is an important issue to study because of its versatility, which controls different cell processes essential for cellular function, including stem cell differentiation. Much evidence points at an important physiological role of extracellular ATP during neuronal development by stimulating proliferation and/or differentiation of NSC and progenitor cells depending on the repertoire of P2 receptor subtype expression. Agonists and antagonists might provide novel and powerful tools for modulating these cell functions for therapy of developmental diseases and regeneration therapy in neurodegenerative diseases. P2X and P2Y purinergic receptors promote proliferation by a mechanism in which ATP induces increases in [Ca^2+^]_i_ in form of waves or spikes, leading to activation of various effectors, followed by an alteration in transcription factor expression and activity patterns such as CREB, NFAT and MEF2, which are involved in stimulation of neural gene transcription. Better understanding of these processes will establish the importance of purinergic signaling in stem cell biology.

## Abbreviations

[Ca^2+^]i: Cytosolic free Ca^2+^ concentration; ADP: Adenosine 5'-diphosphate; AMP: Adenosine monophosphate; ATP: Adenosine 5’-triphosphate; BDNF: Brain derived neurotrophic factor; CAM: Calmodulin; CaMK: Ca^2+^/Calmodulin dependent kinase; CaRE: Calcium response element; CBP: CREB binding protein; Cn: Calcineurin; CREB: Camp/Ca^2+^ response element binding protein; CSC: Embryonal carcinoma cells; ER: Endoplasmic reticulum; hMSC: Adult bone marrow mesenchymal; ICS: Intracellular calcium signaling; IP3: Inositol-1,4,5-Trisphosphate; MEF2: Myocyte enhancer factor-2; NFAT: Nuclear factor of activated T-Cells; nNOS: neuronal nitric oxide synthase; PLC-β: PhospholipaseC-β; ROCs: Receptor-operated channels; RYR: Ryanodine receptor; UDP: Uridine-5'-diphosphate; UTP: Uridine-5'-triphosphate; VOCs: Voltage-operated channels.

## Competing interests

The authors declare to have no competing interests.

## Authors’ contribution

All authors contributed to the writing of the manuscript. Figures 1–3 were designed by T.G. All authors read and approved the final manuscript.
